# Plant Virus and Virus-like Disease Threats to Australia’s North Targeted by the Northern Australia Quarantine Strategy

**DOI:** 10.3390/plants10102175

**Published:** 2021-10-14

**Authors:** Richard I. Davis, Lynne M. Jones, Bradley Pease, Sandy L. Perkins, Harshitsinh R. Vala, Pere Kokoa, Marilyn Apa, Christopher J. Dale

**Affiliations:** 1 Northern Australia Quarantine Strategy, Department of Agriculture Water and Environment, GPO Box 858, Canberra, ACT 2601, Australia; lynne.jones@awe.gov.au (L.M.J.); bradley.pease@awe.gov.au (B.P.); sandy.perkins@awe.gov.au (S.L.P.); harshitsinh.vala@awe.gov.au (H.R.V.); 2 National Agriculture Quarantine and Inspection Authority (NAQIA), P.O. Box 741, Port Moresby 121, Papua New Guinea; pkokoa@naqia.gov.pg (P.K.); mapa@naqia.gov.pg (M.A.); 3 International Plant Health Surveillance Program, Department of Agriculture Water and Environment, GPO Box 858, Canberra, ACT 2601, Australia; chris.dale@awe.gov.au

**Keywords:** biosecurity, surveillance, plant health, virus, phytoplasma, liberibacter

## Abstract

The Northern Australia Quarantine Strategy (NAQS) is a biosecurity initiative operated by the Australian federal government’s Department of Agriculture, Water and the Environment (DAWE). It is unique worldwide because it deals specifically with the potential arrival via unregulated pathways of exotic threats from overseas in a vast and sparsely populated region. It aims to protect the nation’s animal- and plant-based production industries, as well as the environment, from incursions of organisms from countries that lie immediately to the north. These are diseases, pests, and weeds present in these countries that are currently either absent from, or under active containment in, Australia and may arrive by natural or human-assisted means. This review article focuses on the plant viruses and virus-like diseases that are most highly targeted by the NAQS program. It presents eight pathogen species/group entries in the NAQS A list of target pathogens, providing an overview of the historical and current situation, and collates some new data obtained from surveillance activities conducted in northern Australia and collaborative work overseas.

## 1. Introduction

The Australian federal government’s Department of Agriculture, Water and the Environment (DAWE) operates a multi-faceted program called the Northern Australia Quarantine Strategy (NAQS) (https://www.agriculture.gov.au/sites/default/files/documents/naqs_30years_brochure_2020.pdf, accessed on 8 September 2021). NAQS was established in 1989 to specifically address unique plant and animal biosecurity challenges facing Australia’s northern coastline, from Broome in Western Australia to Cairns in Queensland. These arise directly from Australia’s close proximity to Indonesia, Timor-Leste and Papua New Guinea (PNG), which lie to the north and have a dramatically different plant and animal health status to that of Australia. At its closest point, the distance between Australia’s Saibai Island and the coast of PNG is about 4 km.

From a plant health perspective, movement south of insect pests, diseases and weeds can occur naturally, such as with winds created by wet-season monsoon-trough weather systems, or by human-mediated means, such as legal and illegal vessel arrivals. A cornerstone of the NAQS plant health program has been regular surveillance conducted by experienced plant scientists across Australia’s north aiming for early detection of new incursions. This would allow eradication before they reach plant-based industries or threatened populations of plants of environmental significance to the south. Another pillar of the plant health strategy has been collaborative surveys with government biosecurity scientists in these neighboring countries to obtain an ‘over the horizon’ view of current threats. This included several surveys in Indonesia in the 1990’s, including the Indonesian provinces of Papua and West Papua (then known as Irian Jaya), which occupy most of the western half of the island of New Guinea. Annual surveys have also been conducted in PNG and Timor-Leste from the early 1990’s to present. This collaboration continues today under the auspices of DAWE’s International Plant Health Surveillance Program with PNG’s National Agricultural Quarantine and Inspection Authority (NAQIA) and Timor-Leste’s National Directorate for Quarantine and Biosecurity (DNQB). This surveillance relies heavily on target lists of threats. Most are exotic to Australia, although some do occur within the country but are of limited distribution and subject to government biosecurity controls. The highest priority plant disease targets are placed on an A list, and the plant virus and virus-like (phytoplasma, liberibacter and viroid) pathogens from the NAQS A list are presented in Table 1. This paper selects certain key entries in this list and provides some of the background behind them, describes the NAQS program’s involvement with them, and provides an update on the current situation obtained from on-the-ground surveillance activities. When virus and virus-like disease samples are collected for testing in NAQS laboratories in Australia, a standard protocol is used. Approximately 1 g of fresh leaf, shoot or other tissue sample is finely chopped on the day of collection and rapidly desiccated in the field over anhydrous calcium chloride (CaCl_2_) or silica gel at a temperature of 4 °C until fully desiccated, then stored at −20 °C thereafter. Samples collected overseas are subjected to gamma irradiation at 25 kGy upon return to Australia to fulfil import permit requirements.

## 2. *Bean common mosaic virus* (BCMV), Peanut Stripe Strain

### Surveillance to Demonstrate Absence from PNG and North Queensland

The peanut stripe strain of the virus species *Bean common mosaic virus* (BCMV, genus *Potyvirus*) causes peanut stripe disease. This virus, previously known as peanut stripe virus, assumed international biosecurity importance following introduction into the USA from China in 1979 [14]. Southeast Asian strains of this virus are regarded as a major threat to Australia’s peanut production industry as they cause significant losses, with peanut yield losses of over 50% demonstrated in Indonesia [15]. A threat to Australia’s soybean production may also exist as this crop is severely affected by this virus in Taiwan [16]. Australia’s peanut and soybean crops are an important component of the nation’s AUD 11.4 billion grains industry [17]. For several years peanut stripe has been known to be widespread in southeast Asia [18]. It is also widespread in the Indonesian half of the island of New Guinea [2]. This virus is transmitted at a relatively high rate in infected seed, with up to 50% recorded [19]. It is also transmitted by a number of common aphids and its large host range includes cultivated and weedy legumes [20]. These factors combine to greatly increase the likelihood of entry into PNG across the land border from Indonesia. To address this threat, NAQS has implemented an ongoing program of collecting possible symptomatic peanut and other legume leaf samples on surveys conducted both in PNG and Far North Queensland. They have been indexed to determine if they are infected with viruses in the peanut stripe strain group of BCMV. This was done by initial serological screening to test for infection by members of the potyvirus group and in some cases also BCMV. RNA was then extracted from ELISA-positive samples, and also from some samples not initially screened serologically in later years, and subject to a potyvirus group-specific reverse transcriptase polymerase chain reaction (RT-PCR) test. This was followed by DNA sequence and phylogenetic analysis to determine any relationship to the peanut stripe strain of BCMV. So far, 49 legume leaf samples showing virus-like leaf symptoms in PNG and 47 from far north Queensland have been indexed negative in this way (Supplementary Materials Table S1). A spin off from this has become a broader legume potyvirus diversity and distribution study which is currently in progress. 

## 3. *Ramu stunt virus* (RmSV, Provisional Name)

### A Virus That Is Not Ubiquitous across PNG

The provisionally named *Ramu stunt virus* (RmSV), a virus which has homology to the genus *Tenuivirus*, causes Ramu stunt disease of sugarcane, which is unique to PNG. The disease first appeared as a severe epidemic of stunting, yellowing and other symptoms at the Ramu Sugar Plantation in Morobe Province in 1986, with a 40% reduction in total sugarcane production [21]. Up to 30% of all imported varieties tested were susceptible to Ramu stunt [22]. Early work suggesting that a phytoplasma may be involved [23] was dismissed and a consistent association with what appeared to be a unique virus, related to the tenuivirus group, was demonstrated instead [24]. Since then, the full genome (six RNA fragments) of one isolate has been sequenced and the provisional species name ‘Ramu stunt virus’ assigned [25]. The leafhopper, *Eumetopina flavipes*, was implicated as the vector of the disease before etiology was fully understood [26]. Transmission and the causal relationship have now been confirmed by molecular techniques [27]. *E. flavipes* is absent from commercial sugarcane fields in Australia but is common on the Torres Strait Islands and has also been recorded in Queensland’s Northern Peninsula Area [28]. RmSV could be introduced into Australia in illegally imported plants or cuttings. There is also some risk that the virus could be carried into the Torres Strait by its insect vector. True sugarcane (*Saccharum officinarum* rather than commercial *Saccharum* hybrids) is a common food plant in gardens throughout the Torres Strait Islands and northern Australia. This potentially provides an establishment and spread pathway to commercial production areas further south, valued at AUD 1.25 billion in 2019 [17]. Although Ramu stunt disease was once believed to be widespread across PNG based on visual symptoms [29], actual diagnostic testing has so far identified the virus in symptomatic sugarcane collected only from the Ramu valley, Morobe Province and Alotau, Milne Bay Province [7,27]. The diagnostic test, using RT-PCR and primers with homology to tenuivirus RNA, is being used by virologists at the Sugar Research Australia (SRA) biotechnology laboratory in Brisbane [27,30]. NAQS surveys have targeted sugarcane stands showing possible stunting and yellowing. A program of virus indexing of these *S. Officinarum* samples by SRA using these diagnostic tests has so far returned no evidence for the presence of this disease in PNG’s Western Province or the Torres Strait Islands (Table S2). 

## 4. *Cotton leafroll dwarf Virus* (CLRDV)

### An Unexpected Recent Discovery at Australia’s ‘Back Door’

The virus species *Cotton leafroll dwarf virus* (CLRDV, genus: *Polerovirus*) is the causal agent of cotton blue disease. CLRDV is now considered to be synonymous to the previously reported chickpea stunt disease-associated virus (CpSDaV) [5] and both viruses have been reported infecting cotton and chickpea crops next to each other [31]. This virus causes significant yield losses, reaching up to 80% in Brazil [32]. CLRDV is transmitted by aphids, predominantly by the cotton aphid (*Aphis gossypii*), which has a worldwide distribution, and it is not seed- or mechanically transmitted. Reported hosts of CLRDV include the malvaceous species *Gossypium hirsutum*, *Gossypium barbardense*, *Hibiscus sabdariffa* and *Sida acuta* [33]. Although chickpea stunt disease symptoms are only known in chickpea (*Cicer arietum*), known symptomless hosts include several plants in the Amaranthaceae and Fabaceae families [34]. CLDRV has also been detected from *Hibiscus rosa sinensis* (M. Sharman, unpublished data, 2016). Cotton blue disease was believed to be far from Australia until it was reported in Thailand [35] and was then detected in Timor-Leste on a joint NAQS/Timor-Leste Ministry of Agriculture and Fisheries plant health survey [5]. The virus was found in an asymptomatic domestic *G. barbadense* planting. As there is no commercial cotton production in the country, it now remains to be seen if CLRDV from Timor-Leste is capable of causing cotton blue disease in *G. hirsutum*. Simple pathogenicity tests are now needed in Timor-Leste to determine the risk to Australia’s cotton industry, which is currently expanding rapidly in northern regions. If pathogenic, a possible threat to Australia could be via direct introductions of viruliferous vector aphids via wind during the monsoon wet season. Similar insect movements have been postulated to have occurred from Timor-Leste to parts of the Northern Territory and northern Western Australia [36,37]. On arrival, aside from cotton crops, other potential host plants that occur in northern Australia include *G. barbadense*, which is found sporadically in communities as a legacy of historical plantings for cotton production, and *H. rosa sinensis*, which is a very common garden and amenity ornamental. The Mitchell Plateau region in northern Western Australia is also a diversity hotspot for the genus *Gossypium* [38], which may act as perennial hosts for CLRDV. Surveillance in northern Australia must account for cotton bunchy top virus (CBTV), which is also caused by a polerovirus and is present in Australia and produces some similar symptoms [39]. Although specific primers are available for CLRDV [35], diagnostics of NAQS surveillance samples currently relies on generic polerovirus primers and sequence analysis, as this facilitates poleroviruses diversity studies.

## 5. *Banana Bunchy Top Virus* (BBTV)

### Appears to Be on the Island of New Guinea, but Not Found in PNG

The virus species *Banana bunchy top virus* (BBTV, genus *Babuvirus*) has been in Australia since the early 1900’s, where it has caused extensive losses [40], but it is currently present only with very low incidence and under strict quarantine containment in northern NSW and southeast Queensland. In the three countries immediately to the north of Australia (PNG, Timor-Leste and Indonesia), the only records of BBTV are from Indonesia, where it arrived in the late 1970’s [1]. Although bunchy top disease was not found on joint Australian/Indonesian government plant biosecurity surveys of Indonesia’s half of the island of New Guinea conducted in 1997 and 1999 [2], the highly distinctive symptoms have been observed more recently in an Indonesian border region adjacent to PNG, though laboratory confirmation could not be undertaken [41]. As the symptoms are almost unmistakable, PNG is taking this potential new outbreak very seriously. It is also of concern to Australia because it is highly likely that the BBTV present here will be a member of the Asian group of BBTV isolates [42], and therefore different from what is present in Australia, which is part of the South Pacific group of isolates. The principle long-distance mode of movement of this virus is in banana planting material (suckers) transported by people. Once introduced to a new location, however, the virus can spread from the original source with the movement of its insect vector, the banana aphid *Pentalonia nigronervosa*, a species which is already widespread throughout this region [43]. It is possible that spread could also occur if the recently confirmed additional vector, *Pentalonia caladii* [44], were present, but much less is known about this morphologically similar aphid’s distribution. To address this threat in PNG, joint surveys conducted since 2007 have focused on collecting leaf mid-rib samples from banana plants showing any sort of symptoms remotely similar to those of bunchy top disease and serologically testing them for BBTV. These have mostly been just an upright growth habit, and all returned negative results. Twenty-two of these samples were collected from Sandaun Province, nine from the Western Province, which also shares a border with Indonesia, and a further twelve samples were collected from other provinces further to the east (Table S3). 

## 6. *Fiji Disease Virus* (FDV)

### Appears to Be Absent from Parts of PNG Adjacent to Queensland’s Torres Strait Islands 

The virus species *Fiji disease virus* (FDV, genus *Fijivirus*) causes Fiji leaf gall disease in sugarcane. Fiji leaf gall disease has caused devastating losses and has threatened the existence of the sugar industry in areas of Fiji and southern Queensland [45]. Although FDV is present in Australia’s commercial sugarcane, which is grown only in Queensland and northern New South Wales (NSW), it is restricted in distribution. The virus has never been established north of Proserpine in Queensland (latitude 20.4018° S) and strict biosecurity containment legislation and extensive control programs maintain this situation. Currently the disease occurs with a very low incidence and severity between the sunshine coast and Brisbane in Queensland and in northern NSW only, and it is considered to be close to being eradicated (Nicole Thompson, Sugar Research Australia, unpublished data). This virus is reported from several countries in southeast Asia, [46]. As the symptoms are so distinctive, some reports are not verified with laboratory tests, and amongst the three countries immediately to the north of Australia (PNG, Timor-Leste and Indonesia), a record from Indonesia, including from what is today Papua Province, is one of these [47]. In neighboring PNG, however, the disease has long been known to be widespread and molecular diagnostic testing has confirmed FDV [2]. The virus is transmitted in a persistent manner by planthoppers of the genus *Perkinsiella. P. saccharicida* is the only known vector present in Australia and is widespread in commercial cropping areas, and it is also present in PNG [48]. *P. saccharicida* is known to travel long distances, at least 30 km and perhaps up to 400 km [45]. If present nearby, Fiji leaf gall disease could spread to islands in the Torres Strait from Indonesia’s Papua Province or PNG by natural spread of the insect vectors, or with the illegal movement of sugarcane cuttings. Non-commercial sugarcane (*S. officinarum*) is a common food plant in gardens throughout the Torres Strait Islands and northern Queensland. Whilst the vector is present in commercial cropping areas, there are no records from the Torres Strait or Cape York Peninsula (NAQS unpublished data). Although a high incidence of Fiji disease in PNG, ranging from the coastal swamps to the mountainous highlands, has been reported [49], its distribution may be patchy. A series of regular NAQS/NAQIA plant health surveys of PNG’s border regions conducted since 1997 have failed to find symptoms of this disease in the Western Province which lies adjacent to the Torres Strait Islands, and this may lessen the threat of incursion onto islands in the Torres Strait. Meanwhile, numerous visual (NAQS/NAQIA unpublished data) and 12 laboratory test records (Table S4) have confirmed its presence in four other provinces of PNG. The reason for this curious absence from the Western Province remains unknown but may be related to vector and environment because only one collection of a *Perkinsiella* sp. from sugarcane have been made from this province in this series of surveys (NAQIA/NAQS unpublished data). 

## 7. Begomoviruses

### A New and Emerging Threat to Australia’s North

The genus *Begomovirus* (family: *Geminiviridae*) is highly complex, fluid and constantly changing, with many unassigned isolates and numerous sequence variants within individual species [50]; by 2020, there were 445 recognized species [51]. Since the 1980’s, begomoviruses have established themselves as pathogens of escalating importance and increased prevalence worldwide, becoming the main limiting factor in the production of many crops in the tropics and subtropics [52,53]. This has happened mostly as a direct result of the spread of a highly efficient vector insect, the silverleaf whitefly. This vector was originally known as the biotype B of *Bemisia tabaci* and was regarded as part of a large species complex. Today, *B. tabaci* is not considered to be made up of biotypes and is instead a complex of 11 well-defined high-level groups containing at least 24 morphologically indistinguishable species in which this vector is known as *B. tabaci* Middle East-Asia Minor 1 (MEAM1) [54]. The key factors in promoting begomovirus spread were this insect’s broader host range, higher fecundity, increased capacity for dispersal and better ability to develop resistance strategies to insecticidal controls compared to other *B. tabaci* populations [55]. As well as being a more efficient vector, the feeding behavior of this whitefly has facilitated the evolution of new variants of these viruses [56]. Since its first arrival in Australia in 1994 [57], MEAM1 is now widespread in all major tropical and subtropical cropping regions of Australia [58]. Begomovirus-affected crops that are important to the Australian economy include cotton (*G. hirsutum*), tomato (*Solanum lycopersicum*), chili (*Capsicum annuum* var. *annuum*) and sweetpotato (*Ipomoea batatas*). They also infect numerous weeds. In contrast to southeast Asia and India, Oceania has comparatively few records of begomovirus diseases. In important crops, this is tomato yellow leaf curl virus—Israel (TYLCV-IL), which is now considered a ‘severe’ strain of the species *Tomato yellow leaf curl virus* which is present in Queensland [59], New Caledonia [60] and French Polynesia [61]. Also in Australia is the less damaging species *Tomato leaf curl virus* (ToLCV, previously tomato leaf curl virus—Australia), which has been known in the Northern Territory since 1970 and reached parts of Queensland’s Cape York Peninsula in the late 1990s [62]. A novel and as-yet not fully characterized begomovirus has also been found causing severe leaf curl disease in tomatoes in PNG (NAQIA/NAQS/QDAF unpublished data, 2017). There are also records of the species *Sweet potato leaf curl virus* (SPLCV) in Queensland [63], Western Australia and the Northern Territory (NAQS unpublished data), and this virus is also in PNG (NAQIA unpublished data). Novel and as-yet not fully characterized begomoviruses are present at a low incidence in non-crop hosts in PNG (NAQIA/NAQS/QDAF unpublished data). There are also records of begomoviruses in non-crop hosts in Queensland [64]. Unpublished data from Timor-Leste suggests this country has a greater diversity of begomoviruses, similar to others in southeast Asia such as neighboring Indonesia [65]. Here, novel and as-yet not fully characterized begomoviruses are widespread in various crop and non-crop hosts (DNQB/NAQS/QDAF unpublished data). Whiteflies are not efficient flyers but have been known to travel long distances with wind and convection currents. It has been speculated that movement of viruliferous whiteflies on prevailing winds over large sea distances established disease epidemics caused by the same begomoviruses in the Dominican Region and Jamaica [66] and in Puerto Rico and Martinique [67]. It may therefore be possible for direct movement of viable and viruliferous whiteflies on seasonal winds from Timor-Leste to the Northern Territory and from PNG to north Queensland. Viruliferous whiteflies could also enter the country on plant material. There is also a risk that these viruses could enter via fruit imports because the movement of TYLCV in infected tomato fruit followed by whitefly feeding and successful transmission to tomato plants has been demonstrated [68]. Begomoviruses remain a major threat to Australia because of the low diversity of species already present. Introduction of new begomoviruses would not only introduce a new disease but also would potentially increase species and/or strain diversity. This is because recombination occurs readily when several different begomoviruses with common hosts co-exist together, leading to new strains and species [69]. Surveillance for begomoviruses relies on visual inspection for symptoms of begomovirus infection, which are very similar to those of nutrient and other stresses, sampling and molecular diagnostics. DNA extracts can be indexed for begomovirus by PCR using the primer pairs Avcore/Accore [70,71] and SPG1/SPG2 [72]. DNA sequence analysis can then be conducted on positives to indicate the likely identity of the virus. A complete identification may rely on obtaining the sequence of the whole DNA-A component using rolling circle amplification (RCA) and also determining satellite DNA sequences.

## 8. ‘*Candidatus Liberibacter asiaticus*’

### A History of Monitoring on the Island of New Guinea

HLB is regarded as the worst diseases affecting citrus trees and is caused by the phloem-limited unculturable bacterium ‘*Candidatus Liberibacter asiaticus*’. ‘*Ca.* L. asiaticus’ is vectored by the Asian citrus psyllid (ACP) *Diaphorina citri*, and long distance spread occurs via infected and infested planting material. Since the first detection of ACP in the northwest of Indonesia’s West Papua Province (then Irian Jaya) in 1990 [73], NAQS surveillance has targeted both the insect and the disease on the island of New Guinea. ACP was then recorded over 1000 km to the east, near Jayapura, in 1992 (NAQS unpublished data 1992). NAQS surveillance then provided the first verification of the presence of HLB on the island of New Guinea, firstly in Indonesia’s territory in 1999 [74], then at Vanimo in Sandaun Province of PNG in 2002 [13]. From early 2003, a campaign of quarantine containment, including legislative measures, and public awareness was carried out across PNG. Importantly, delimiting surveys at two months, one year and three years post-incursion indicated the great success of this campaign [75]. These surveys documented little movement of *D. citri* and highly limited disease focus expansion, only in and around the town of Vanimo. Since then, general surveillance has also prioritized this disease, and a body of additional data supports the claim that the distribution of this disease remains highly restricted. By 2021, 107 citrus trees had been sampled because they were showing possible HLB-like symptoms, which are similar to nutrient deficiencies and other stresses. All indexed negative in Australia for the disease in molecular testing (Table S5). Eighty-two of these were citrus trees in Sandaun Province, 12 in the Western Province, 11 in East Sepik Province, two in the Eastern Highlands Province and one in each of Morobe Province, the Autonomous Region of Bougainville, Chimbu Province, Jiwaka Province, Milne Bay Province and the Southern Highlands Province. Currently, the only records of HLB are still confined to the Vanimo area, where the disease remains hard to find after affected trees died or were destroyed by owners as they became unproductive. The success of this containment is related to several factors. Within the affected region, the landscape is peri-urban, with no commercial citrus orchards, and marcotting is not commonly practiced. Further geographical spread may have been prevented because there are no reliable road linkages to the rest of the country. However, perhaps the most important component of this story of success relates to the psyllid vectors. The ACP population crashed down to very low numbers in the late 2000s, around eight years after the outbreak (NAQIA/NAQS unpublished data), and investigations are ongoing into the possible causes of this.

## 9. Phytoplasmas Affecting Palms and Banana Plants

### A Group of Related Pathogens That Needs Further Characterisation

Phytoplasmas are similar to ‘*Candidatus* Liberibacter’ in that they are also unculturable, phloem-limited bacteria that can survive and multiply only in the phloem vessels of their host plants and their insect vectors. A key difference is phytoplasma’s lack of a cell wall. Phytoplasma classification currently uses 16S rRNA gene sequences to assign isolates to ‘*Candidatus* Phytoplasma’ species based upon the percentage sequence identity of the 16S rRNA gene [76]. Restriction fragment length polymorphism (RFLP) patterns of the 16Sr gene, now usually done in silico (https://plantpathology.ba.ars.usda.gov/phytoplasma.html, accessed on 8 September 2021), are used to assign phytoplasmas to known 16Sr groups and subgroups. In PNG, a serious wilt disease among cooking banana plants (ABB genomic group) associated with phytoplasmas was first described in 2012, and the phytoplasmas involved were named banana wilt-associated phytoplasmas (BWAP) [11]. This was shortly after a phytoplasma associated with a devastating lethal wilt disease of coconut palms (*Cocos nucifera*), known as Bogia coconut syndrome (BCS), was first associated with a phytoplasma in the Bogia district of PNG’s Madang Province [77]. Later, BCS and BWAP phytoplasmas from that region of the country were shown to be sufficiently similar to each other to be combined in a novel taxon, ‘*Candidatus* Phytoplasma noviguineense’ [12]. However, more variation exists within the group of closely related phytoplasmas causing wilt symptoms in cooking banana plants. In published phylogenetic analyses [78,79], it can be seen that BWAPs from the Autonomous Region of Bougainville and the Solomon Islands separate out from members of ‘*Ca.* P. noviguineense’ from PNG’s northwest. It is possible that they represent another novel ‘*Candidatus* Phytoplasma’ taxon. When these phytoplasmas (from both east and west) were analyzed using the *i*Phyclassifier program [80], they could not be assigned to any previously established 16Sr groups/subgroups [78]. The closest matches (with similarity coefficients below the group/subgroup assignment cut-off point) were to phytoplasmas affecting palms elsewhere in the world that belong to groups 16SrIV and 16Sr XXII. The potential vectors and alternative hosts of ‘*Ca.* P. noviguineense’ have been studied extensively by an Australian Centre for International Agricultural Research project in the region in recent years [81]. These studies used artificial feeding medium experiments to identify the planthoppers *Zophiuma pupillata* and *Lophops saccharicida* as species of particular importance [82]. Subsequently, field-collected individuals of both these two species were capable of transmitting phytoplasma to banana plants in caged transmission trials [81]. The BCS project also identified betel nut palms (*Areca catechu*) and taro (*Colocasia esculenta*) as key alternative hosts using nested PCR and sequence analysis [81]. 

Losses of both cooking banana plants and coconut palms have been extensive, reaching thousands of plant deaths. To date, there have been no reports of a BCS-like disease epidemic from any areas outside of Madang Province. This has been backed up by opportunistic sampling and molecular testing of palms encountered on plant health surveys conducted by NAQIA/NAQS. This has targeted palms found showing any symptoms remotely similar to those of BCS since the outbreak started in Bogia District. A total of 65 palms have been indexed negative for phytoplasma (Table S6). These comprise 14 palms in West New Britain Province, 11 in the Western Province, 8 in East Sepik Province, 6 in the Autonomous Region of Bougainville and Central Province, 4 in Sandaun Province, 2 in Morobe Province and 1 in New Ireland Province (plus 10 from investigations made in zones otherwise considered disease-free in Madang Province). Also of great importance is the fact that banana plants and coconut palms are dying together only in Madang Province. Elsewhere in PNG and the Solomon Islands, only the banana plants are affected (NAQS/NAQIA/Biosecurity Solomon Islands unpublished data). This may be because of key pathogenic variation within this closely related group of phytoplasmas. It is unlikely that these differences are related to vector insect distributions because the suspected vectors of ‘*Ca*. P. noviguineense’ are likely to be widespread across the country [82]. Another possible explanation may be that the latent period between infection and symptom expression in palms is much longer than in banana plants. However, field observations over a period of more than ten years in PNG suggest this is not the case (NAQS/NAQIA unpublished data). 

## 10. Conclusions

The case histories outlined here are a testament to the great value of inter-agency and inter-governmental collaboration. Whilst conducting ‘over the horizon’ surveillance for Australia, mutually beneficial biosecurity outcomes are achieved for Australia and partner countries. Half of the virus/virus-like disease threats discussed here are new to the region, having arrived from southeast Asia, and two others probably originated on the island of New Guinea. It is valuable to raise awareness of these diseases which not only pose a potential biosecurity risk to Australia but also to other island nations further to the east in the Pacific.

## Figures and Tables

**Table 1:** NAQS A list target viruses and virus-like diseases.

Species	Genus	Disease Common Name	Major Commodity Affected	Principal Insect Vector(s)	Nearest Published Location to Northern Australia
Viruses					
*Banana bunchy top virus*	*Babuvirus*	Banana bunchy top	Banana	Aphids (*Pentalonia* spp.)	Indonesia ^A^ [1]
*Bean common mosaic virus* (peanut stripe strain)	*Potyvirus*	Peanut stripe	Peanuts, soybean	Aphids (several spp.)	Indonesia’s Papua province [2]
Many species and strains	*Begomovirus*	Many names	Crops in the Cucurbitaceae, Solanaceae, Fabaceae and Malvaceae	Whiteflies (*Bemisa tabaci*)	Indonesia [3]
*Chilli veinal mottle virus*	*Potyvirus*	None	Chilli, capsicum, tomato	Aphids (several spp.)	Indonesia’s Papua province [2]
*Citrus tristeza virus* (exotic strains)	*Closterovirus*	Tristeza	Citrus	Aphids (*Toxoptera citricida*)	Indonesia [4]
*Cotton leafroll dwarf virus*	*Polerovirus*	Cotton blue disease	Cotton	Aphids (*Aphis gossypii*)	Timor-Leste [5]
*Fiji disease virus*	*Fijivirus*	Fiji disease	Sugarcane	Planthoppers (*Perkinsiella* spp.)	PNG ^A^ [2]
*Papaya ringspot virus* (papaya infecting strain)	*Potyvirus*	Ringspot	Papaya	Aphids (several spp.)	Indonesia [6]
*Ramu stunt virus* (provisional name)	*Tenuivirus*	Ramu stunt	Sugarcane	Leafhoppers (*Eumetopina flavipes*)	PNG [7]
*Sorghum mosaic virus* (exotic strains)	*Potyvirus*	Sorghum mosaic	Sorghum, maize	Aphids (several spp.)	Philippines [8]
*Sugarcane mosaic virus* (exotic strains)	*Potyvirus*	Sugarcane mosaic	Sugarcane	Aphids (several spp.)	Indonesia [8]
*Sugarcane streak mosaic virus*	*Poacevirus*	Sugarcane streak mosaic	Sugarcane	Aphids (identity uncertain)	Indonesia [9]
**Virus-like**					
Tomato apical stunt viroid	*Pospiviroid*	None	Tomato, potato	Bees (*Bombus terrestris*)	Indonesia [10]
Banana wilt-associated phytoplasmas	‘*Candidatus* Phytoplasma’	Lethal wilt	Banana	Unknown	PNG [11]
‘*Candidatus* Phytoplasma noviguineense’	‘*Candidatus* Phytoplasma’	Bogia coconut syndrome	Banana, palms	Unknown	PNG [12]
‘*Candidatus Liberibacter asiaticus*’	‘*Candidatus* Liberibacter’	Huanglongbing (HLB)	Citrus	Asian citrus psyllid (*Diaphorina citri*)	PNG [13]

^A^ Also present, but under biosecurity containment, in a restricted area within Australia south of the NAQS region.

## Supplementary Materials

The following are available online at https://www.mdpi.com/article/10.3390/plants10102175/s1, Table S1: Legume samples collected and indexed negative for the peanut stripe strain of *Bean common mosaic virus* in PNG and Far North Queensland, Australia, Table S2: Sugarcane samples indexed negative for the *Ramu stunt virus*, Table S3: Banana leaf samples collected on PNG surveys and indexed negative for *Banana bunchy top virus*, Table S4: Sugarcane samples indexed positive for *Fiji leaf gall disease virus*, Table S5: Negative HLB PCR test records from PNG, Table S6: Palm samples collected from outside known BCS-affected areas that indexed negative for phytoplasma in PCR testing.
