# Supplementary material for: Plant Virus and Virus-like Disease Threats to Australia’s North Targeted by the Northern Australia Quarantine Strategy
Source: Plants (Basel). 2021 Oct 14;10(10):2175. doi: 10.3390/plants10102175 (PMC8537380; doi:10.3390/plants10102175)
Supplement: Supplementary file 1 [file plants-10-02175-s001.zip › Supplementary tables.pdf]

plants MDPI

Supplementary tables

Table S1. Legume samples collected and indexed negative for the peanut stripe strain of bean common mosaic virus in PNG and Far North Queensland, Australia

Collection No.	Legume	Symptoms[A]	Location[B]	Potyvirus ELISA[C]	BCMV ELISA[D]	RT-PCR/ sequence analysis[E]
			**PNG**			
MPW1092	*Vigna unguiculata* ssp. *sesquipedalis*	YOGM	Kiunga, WP	-	NT	NT
MPW1024	*Arachis hypogaea*	Chlorotic mottle	Daru, WP	-	NT	NT
MPW1044	*Arachis hypogaea*	Chlorotic mottle	Nomad River, WP	-	NT	NT
MPW1045	*Arachis hypogaea*	Chlorotic mottle	Nomad River, WP	-	NT	NT
RID5119	*Calopogonium mucunoides*	YOGM	Tiomini, Kiunga, WP	-	NT	NT
RID5120	*Calopogonium mucunoides*	YOGM	Tiomini, Kiunga, WP	-	NT	NT
RID5036	*Centrosema molle*	YOGM	Daru, WP	-	NT	NT
RID5040	*Centrosema molle*	YOGM	Daru, WP	-	NT	NT
RID5129	*Arachis hypogaea*	Chlorotic treaks	Debapari, WP	-	NT	NT
RID5160	*Arachis hypogaea*	Chlorotic marking	Ningerum, WP	-	NT	NT
RID5436	*Calopogonium mucunoides*	YOGM	Somboi, SP	-	NT	NT
RID5471	*Calopogonium mucunoides*	YOGM	Blackwater, SP	-	NT	NT
RID5494	*Calopogonium mucunoides*	YOGM	Vanimo, SP	-	NT	NT
RID5501	*Calopogonium mucunoides*	YOGM	Lowan, ESP	-	NT	NT
RID5560	*Centrosema molle*	YOGM	Zenag, MoP	-	NT	NT
RID5577	*Centrosema molle*	YOGM	Ramu Valley, MoP	-	NT	NT
RID6619	*Arachis hypogaea*	Chlorotic marking	Aiyura, EHP	-	-	NT
RID6620	*Arachis hypogaea*	Chlorotic	Aiyura, EHP	-	-	NT

		marking				
RID6626	*Arachis hypogaea*	Chlorotic striping	Bena, EHP	-	-	NT
RID6634	*Arachis hypogaea*	Chlorotic striping	Okiufa, EHP	-	-	NT
RID6643	*Arachis hypogaea*	Chlorotic striping	Asaro River, EHP	-	-	NT
RID6646	*Crotalaria micans*	Stripey YOGM	Komunive, EHP	+	+	Not peanut stripe group[F]
RID6664	*Crotalaria micans*	YOGM / mottle	Kundiawa, CP	+	+	Not peanut stripe group[F]
RID6665	*Crotalaria micans*	YOGM / GOYVB	Kundiawa, CP	+	+	Not peanut stripe group[F]
RID6666	*Arachis hypogaea*	Chlorotic marking	Kundiawa, CP	-	-	NT
RID6702	*Arachis hypogaea*	Chlorotic mottle	Gumanch, WHP	-	-	NT
RID7009	*Phaseolus vulgaris*	Chlorotic marking	Tabubil, WP	-	NT	NT
RID7014	*Arachis hypogaea*	GOYVB	Tabubil, WP	-	NT	NT
RID7027	*Arachis hypogaea*	Chlorotic spots	Ningerum, WP	-	NT	NT
RID7029	*Arachis hypogaea*	Chlorotic spots	Ningerum, WP	-	NT	NT
RID7033	*Arachis hypogaea*	Chlorotic spots	Kiunga, WP	-	NT	NT
RID7040	*Centrosema molle*	YOGM	Kiunga, WP	-	NT	NT
RID7051	*Vigna unguiculata* ssp. *sesquipedalis*	YOGM	Debepari, WP	-	NT	NT
RID7053	*Vigna unguiculata* ssp. *sesquipedalis*	Chlorotic spots	Morehead, WP	-	NT	NT
RID7066	*Centrosema molle*	Chlorotic marking	Daru, WP	-	NT	NT
RID7079	*Arachis hypogaea*	Chlorotic spots	Daru, WP	+m	-	NT
RID7743	*Desmodium* sp.	YOGM, crinkle	Bougainville Is. AROB	NT	NT	Unrelated potyvirus[G]
RID7761	*Arachis hypogaea*	Chlorosis	Wewak, ESP	-	NT	NT
RID8030	*Arachis hypogaea*	YOGM, stripes	Lae, MoP	-	NT	NT
RID8040	*Arachis hypogaea*	YOGM	Lae, MoP	-	NT	NT
RID8048	*Arachis hypogaea*	YOGVB	Lae, MoP	-	NT	NT

RID8062	*Vigna unguiculata* ssp. *sesquipedalis*	YOGM	Port Moresby, NCD	+	NT	Not peanut stripe group[F]
LMJ1081	*Vigna unguiculata ssp. sesquipedalis*	YOGM	Daru, WP	NT	NT	Unrelated potyvirus[G]
LMJ1224	*Vigna unguiculata ssp. sesquipedalis*	YOGM	Bougainville Is. AROB	NT	NT	Not peanut stripe group[F]
LMJ1233	*Crotolaria* sp.	YOGM	Bougainville Is. AROB	NT	NT	Unrelated potyvirus[G]
LMJ1250	*Vigna unguiculata ssp. sesquipedalis*	YOGM	Bougainville Is. AROB	NT	NT	Not peanut stripe group[F]
LMJ1259	*Vigna unguiculata* ssp. *sesquipedalis*	YOGM	Bougainville Is. AROB	+	NT	Not peanut stripe group[F]
LMJ1262	*Calopogonium mucunoides*	N/A	Bougainville Is. AROB	NT	NT	Unrelated potyvirus[G]
MA106	*Vigna unguiculata ssp. sesquipedalis*	NA	Old Mawatta, WP	NT	NT	Unrelated potyvirus[G]
			**Queensland**			
LMJ254	*Clitoria ternatea*	Chlorosis, crinkle	Boigu Is., TS	+	+	Not peanut stripe group[F]
LMJ396	*Calopogonium mucunoides*	YOGM	Aurukun, CYP	-	NT	NT
RID6404	*Calopogonium mucunoides*	YOGM	Weipa, CYP	-	NT	NT
LMJ453	*Clitoria ternatea*	YOGM	Boigu Is., TS	+	+	Not peanut stripe group[F]
RID6559	*Vigna unguiculata* ssp. *sesquipedalis*	YOGM	Thursday Is., TS	-	NT	NT
LMJ487	*Arachis pintoi*	YOGM	Cooktown, CYP	+m	+	Not peanut stripe group[F]
RID6576	*Chamaecrista rotundifolia*	YOGM	New Mapoon, NPA	-	-	NT
RID6579	*Flemingia parviflora*	YOGM	Bamaga, NPA	-	-	NT
RID6592	*Pueraria lobata*	YOGM	Mer Is., TS	-	-	NT
RID6604	*Desmodium tortuosum*	YOGM	Ugar Is., TS	+	+	Not peanut stripe group[F]
RID6614	*Clitoria ternatea*	YOGM	Kerriri Is., TS	+	+	Not peanut stripe group[F]
RID6947	*Crotalaria goreencis*	YOGM	Erub Is., TS	+	+	Unrelated potyvirus[G]
RID6965	*Chamaecrista rotundifolia*	YOGM	Bamaga, NPA	+	+	Not peanut stripe group[F]

RID6970	*Vigna unguiculata* ssp. *sesquipedalis*	YOGM	Injinoo, NPA	-	NT	NT
LMJ540	*Chamaecrista rotundifolia*	Chlorotic marking	Mapoon, CYP	-	NT	NT
LMJ542	*Chamaecrista rotundifolia*	YOGM	Mapoon, CYP	-	NT	NT
LMJ547	*Calopogonium mucunoides*	Mosaic	Weipa, CYP	-	NT	NT
LMJ700	*Vigna unguiculata* ssp. *unguiculata*	YOGM	Endeavour valley, CYP	NT	NT	Unrelated potyvirus[G]
LMJ701	*Vigna unguiculata* ssp. *unguiculata*	YOGM	Endeavour valley, CYP	NT	NT	Unrelated potyvirus[G]
LMJ704	*Chamaecrista rotundifolia*	YOGM	Bamaga, NPA	NT	NT	-
LMJ707	*Chamaecrista rotundifolia*	YOGM	Bamaga, NPA	NT	NT	-
LMJ711	*Chamaecrista rotundifolia*	YOGM	Bamaga, NPA	NT	NT	Unrelated potyvirus[G]
LMJ715	*Vigna unguiculata sesquipedalis*	YOGM	Bamaga, NPA	NT	NT	Unrelated potyvirus[G]
LMJ716	*Chamaecrista rotundifolia*	YOGM	Bamaga, NPA	NT	NT	Unrelated potyvirus[G]
LMJ722	*Vigna unguiculata sesquipedalis*	Mild mosaic	Seisia, NPA	NT	NT	Unrelated potyvirus[G]
LMJ737	*Chamaecrista rotundifolia*	Mild mosaic	Old Mapoon, CYP	NT	NT	-
LMJ841	*Vigna unguiculata* ssp. *sesquipedalis*	YOGM, distortion	Thursday Is., TS	NT	NT	Unrelated potyvirus[G]
LMJ845	*Clitoria ternatea*	YOGM	Erub Is., TS	NT	NT	Unrelated potyvirus[G]
LMJ848	*Macroptilium atropurpureum*	YOGM	Kerriri Is. TS	NT	NT	Unrelated potyvirus[G]
LMJ858	*Desmodium scorpiurus*	Vein banding	Ugar Is., TS	NT	NT	Unrelated potyvirus[G]
LMJ863	*Clitoria ternatea*	Mild mosaic	Boigu Is., TS	NT	NT	Unrelated potyvirus[G]
LMJ865	*Desmodium scorpiurus*	YOGM	Erub Is., TS	NT	NT	Unrelated potyvirus[G]
LMJ868	*Macroptilium atropurpureum*	YOGM	Erub Is. TS	NT	NT	Unrelated potyvirus[G]
LMJ882	*Clitoria ternatea*	YOGM	Iama Is., TS	NT	NT	Unrelated potyvirus[G]

LMJ916	*Chamaecrista rotundifolia*	Vein banding / mosaic	Bamaga, NPA	NT	NT	-
LMJ1388	*Desmodium tortuosum*	Vein clearing	Warraber Is. TS	-	NT	NT
LMJ1416	*Chamaecrista rotundifolia*	YOGM	Horn Is. TS	NT	NT	-
SLP100	*Vigna unguiculata* ssp. *sesquipedalis*	Light mosaic	Bamaga, NPA	-	NT	NT
SLP180	*Centrosema molle*	YOGM	Weipa, CYP	NT	NT	Unrelated potyvirus[G]
RID7188	*Desmodium tortuosum*	YOGM	Iama Is., TS	NT	NT	Unrelated potyvirus[G]
RID7205	*Erythrina insularis*	Downward leaf curl	Warraber Is., TS	NT	NT	-
RID7927	*Crotalaria goreencis*	YOGM	Kerriri Is., TS	+	NT	Unrelated potyvirus[G]
RID8208	*Clitoria ternatea*	YOGM	Mabuiag Is., TS	NT	NT	Unrelated potyvirus[G]
LMJ1401	*Clitoria ternatea*	YOGM	Erub Is., TS	NT	NT	Unrelated potyvirus[G]
LMJ1404	*Macroptilium atropurpureum*	YOGM	Erub Is. TS	NT	NT	Unrelated potyvirus[G]
LMJ1411	*Centrosema molle*	YOGM, distortion	Thursday Is., TS	NT	NT	Unrelated potyvirus[G]
LMJ1426	*Clitoria ternatea*	Vein feathering	Boigu Is., TS	NT	NT	Unrelated potyvirus[G]

[A]GOYVB: green on yellow vein banding, YOGM: yellow on green mosaic.

[B] AROB: Autonomous Region of Bougainville, CP: Chimbu Province, CYP: Cape York Peninsula, EHP: Eastern Highlands Province, ESP: East Sepik Province, MoP: Morobe Province, NCD: National Capital District, NPA: Northern Peninsula Area (of Cape York Peninsula), SP: Sandaun Province, TS: Torres Strait, WHP: Western Highlands Province, WP: Western Province.

[C]Leaf samples were screened by Plate Trapped Antigen Enzyme Linked Immunosorbent Assay (PTA-ELISA) [1] using Agdia (Elkhart Inc., USA) reagent sets for the potyvirus group. ELISA test results were considered positive (+) when absorbance readings (405nm) exceeded 3 x mean of healthy controls. ELISA test results were considered marginal positive (+m) when absorbance readings (405nm) were less than 3 x mean of healthy controls but exceeded 2 x mean of healthy controls.

[D]Leaf samples were screened by Plate Trapped Antigen Enzyme Linked Immunosorbent Assay (PTA-ELISA) [1] using Agdia (Elkhart Inc., USA) reagent sets for *Bean common mosaic virus* (BCMV). ELISA test results were considered positive (+) when absorbance readings (405nm) exceeded 3 x mean of healthy controls. NT: not tested.

[E] RNA extracts were used in potyvirus group specific Reverse Transcriptase Polymerase Chain Reactions (RT-PCRs) using the Qiagen One-Step RT-PCR Kit and primer pair MJ1/MJ2 [2] or primer pair MJ1/Poty1 [3]. PCR products of the

expected size were directly sequenced by the Australian Genome Research Facility and the resulting sequences were used for phylogenetic analysis. Some samples tested negative (-) by PCR only, and were not subject to serological testing.
[F]Bean common mosaic virus isolates that did not fall into the peanut stripe group when subjected to phylogenetic analysis, further identification work still in progress.
[G]Potyviruses that did not belong to the virus species, *Bean common mosaic virus* when subjected to phylogenetic analysis, further characterisation still in progress.

Table S2. Sugarcane samples indexed negative for the Ramu stunt virus

Collection number	Approximate date	Approximate location [A]	Symptoms
		**PNG**	
RID5032	June 2008	Daru, WP	Chlorotic mottle
RID5041	June 2008	Daru, WP	Strong chlorotic streaks at margins
RID5043	June 2008	Daru, WP	Strong chlorotic streaks
RID5076	June 2008	Suki, WP	Diffuse chlorotic streaks
RID5910	Nov. 2010	Bougainville, AROB	Fiji leaf gall only[B]
RID5882	Nov. 2010	Buka, AROB	Fiji leaf gall only
RID5883	Nov. 2010	Buka, AROB	Fiji leaf gall only
RID5926	Nov. 2010	Huris, Buin, Bougainville, AROB	Chlorosis, stunt
RID5927	Nov. 2010	Buin, Bougainville, AROB	Fiji leaf gall only
RID5957	Nov. 2010	Nr Kieta, Bougainville, AROB	Fiji leaf gall only
RID6137	June 2011	Goroka, EHP	Mosaic, blotch, galls
RID6138	June 2011	Goroka, EHP	Fiji leaf gall only
RID6157	June 2011	Minj, WHP	Fiji leaf gall only
RID6198	June 2011	Margarima Station, SHP	Chlorosis, stunt
MPW807	Sept. 2002	Kobremnim, SP	Galls
RID6457	Oct. 2012	Aitape, SP	Fiji leaf gall only
RID6488	Nov. 2012	Green River, SP	Chlorosis and stunt
RID6499	Nov. 2012	Amanab, SP	Fiji leaf gall only
RID6624	May 2014	Aiyura, EHP	Fiji leaf gall only
RID6640	May 2014	Osaro River, EHP	Fiji leaf gall only
RID7028	May 2015	Ningerum, WP	Chlorotic streaks
RID7065	May 2015	Daru, WP	Chlorotic streaks
RID7076	May 2015	Daru, WP	Stunt
		**Torres Strait**	
LMJ115	June 2007	Dauan Is.	Mosaic
LMJ117	June 2007	Yorke Is.	Chlorotic streaks
RID5203	Aug. 2008	Boigu Is.	General stunting
RID5280	Feb. 2009	Badu Is.	None
RID5297	March 2009	Dauan Is.	Slight stunt
RID5303	March 2009	Saibai Is.	Slight stunt
RID5661	March 2010	Dauan Is.	Chlorotic blotch
RID5683	March 2010	Coconut Is.	Chimaera –like streaks
RID5688	March 2010	Moa Is.	Yellow on green mosaic
RID5805	June 2010	Boigu Is.	Chlorotic flecks
LMJ359	March 2011	Boigu Is.	Puckering

RID6243	Aug. 2011	Coconut Is.	Slight chlorotic streak
RID6245	Aug. 2011	Warraber Is.	Chlorotic streak, stunt
		**Mainland Qld**	
RID5690	April 2010	Bamaga, NPA, CYP	Chlorotic blotch
LMJ469	March 2013	Aytonvale, CYP,	Pokkah Boeng
RID6980	April 2015	Bamaga, NPA, CYP	Chimaera chlorotic streaks

Leaf samples were tested using RT-PCR and primers with homology to tenuivirus RNA [27, 30] by Sugar Research Australia, Indooroopilly, Queensland, Australia.

[A] AROB: Autonomous region of Bougainville, CYP: Cape York Peninsula, EHP: Eastern Highlands Province, NPA: Northern Peninsula Area, SHP: Southern Highlands Province, SP: Sandaun Province, WHP: Western Highlands Province, WP: Western Province.

Table S3. Banana leaf samples collected on PNG surveys and indexed negative for banana bunchy top virus

Collection number	Approx. date	Genome group	Approximate Location [A]	Symptoms
RID 4800	Oct. 2007	AAA	Wewak, ESP	Upright growth habit
RID 4863	Oct. 2007	AAB	Waininge, SP	Upright growth habit
RID 5083	June 2008	AAB	Suki, WP	Upright growth habit
RID 5107	June 2008	ABB	Tarakbits, WP	Upright growth habit
RID 5409	June 2009	AAA	Vanimo, SP	Upright growth habit
RID 5415	June 2009	AAB	Wutung, SP	Upright growth habit
RID 5416	June 2009	AAB	Wutung, SP	Upright growth habit
RID 5427	June 2009	AAB	Wutung, SP	Upright growth habit
RID 5441	June 2009	AAB	Somboi, Bewani, SP	Upright growth habit
RID 5443	June 2009	AAA	Skotio, Bewani, SP	Upright growth habit
RID 5452	June 2009	AAB	Green River, SP	Upright growth habit
RID 5509	June 2009	AAA	Yangora, ESP	Upright growth habit
RID 6294	Oct. 2011	AAA	Sesareme, WP	Upright growth habit
RID 6297	Oct. 2011	AAA	Kamusi, WP	Slight upright growth habit
RID 6275	Oct. 2011	AAA	Lake Murray, WP	Chlorosis and upright habit
RID 6284	Oct. 2011	AAA	Wereave, WP	Chlorosis and upright habit
RID 6285	Oct. 2011	ABB	Tambarai, WP	Slight upright growth habit
RID 6289	Oct. 2011	ABB	Balamuk, WP	Slight upright growth habit
RID 6456	Oct. 2012	ABB	Poro, SP	Slight upright growth habit
RID 6468	Oct. 2012	ABB	Nr Poin, SP	Slight upright growth habit
RID 6470	Oct. 2012	ABB	Wutung, SP	Slight upright growth habit
RID 6476	Oct. 2012	AAA	Ituli, SP	Slight upright growth habit
RID 6478	Oct. 2012	AAA	Skotio, SP	Slight upright growth habit
RID 6487	Oct. 2012	AAB	Green River, SP	Slight upright growth habit
RID 6493	Oct. 2012	AAA	Yapsi, SP	Slight upright growth habit
RID 6496	Oct. 2012	AAB	Amanab, SP	Slight upright growth habit
RID 6709	May 2014	ABB	Mt Hagen, WHP	Slight upright growth habit
RID 6691	May 2014	ABB	Banz, JP	Slight upright growth habit
RID 6679	May 2014	ABB	Minj, JP	Slight upright growth habit
RID 6674	May 2014	AAA	Kebamuk, CP	Slight upright growth habit
RID 6658	May 2014	AAA	Mt Wilhelm, CP	Slight upright growth habit
RID 6641	May 2014	AAB	Asaro River EHP	Slight upright growth habit
RID 7011	May 2015	AAA	Tabubil, WP	Slight upright growth habit
RID 7263	April 2016	AAA	Vanimo, SP	Slight upright growth habit
RID 7269	April 2016	AAA	Musu, SP	Slight upright growth habit
RID7274B	April 2016	AAA	Lido, SP	Slight upright growth habit
RID 7293	April 2016	ABB	Imbine, SP	Slight upright growth habit

RID 7294	April 2016	ABB	Imbine, SP	Slight upright growth habit
RID 7300	April 2016	AAA	Skotiaoh SP	Slight upright growth habit
RID 7741	Nov. 2017	ABB	Dugur, ESP	Slight upright growth habit
RID 7750	Nov. 2017	AAB	Moem, ESP	Slight upright growth habit
RID 7772	Nov. 2017	ABB	Wewak, ESP	Choke throat
MA104	Nov. 2017	AAB	Old Mawatta, WP	NA

All samples tested negative by double antibody sandwich enzyme linked immunosassay (DAS-ELISA) using Agdia (Elkhart Inc., USA) reagent sets and procedures and chemical preparations similar to those described in[4] for banana bunchy top virus (BBTV).

[A] EHP: Eastern Highlands Province, ESP: East Sepik Province, JP: Jiwaka Province, SP: Sandaun Province, CP: Chimbu Province, WP: Western Province, WHP: Western Highlands Province.

Table S4. Sugarcane samples indexed positive for Fiji leaf gall disease virus

Collection number	Approximate date	Approximate location [A]
MPW807	Sept. 2002	Kobremnim, SP
RID5910	Nov. 2010	Bougainville, AROB
RID5882	Nov. 2010	Buka, AROB
RID5883	Nov. 2010	Buka, AROB
RID5927	Nov. 2010	Buin, Bougainville, AROB
RID5957	Nov. 2010	Nr Kieta, Bougainville, AROB
RID6138	June 2011	Goroka, EHP
RID6157	June 2011	Minj, WHP
RID6457	Oct. 2012	Aitape, SP
RID6499	Nov. 2012	Amanab, SP
RID6624	May 2014	Aiyura, EHP
RID6640	May 2014	Osaro River, EHP

Leaf samples were tested using RT-PCR modified from [5] by Sugar Research Australia, Indooroopilly, Queensland, Australia.

[A]AROB: Autonomous Region of Bougainville, EHP: Eastern Highlands Province, SP: Sandaun Province, WHP: Western Highlands Province.

Table S5. Negative HLB PCR test records from PNG

Collection No.	Date	Location[A]	Latitude	Longitude	Tree identity
MPW1273	Sept. 2004	Wutung, SP	-2.61165	141.0121	*Citrus sp.*
MPW1270	Sept. 2004	Hap Hap, SP	-2.72305	141.2652	*Citrus maxima*
MPW1271	Sept. 2004	Wutung, SP	-2.60828	141.0045	*Citrus reticulata*
MPW1272	Sept. 2004	Wutung, SP	-2.60828	141.0045	*Citrus reticulata*
MPW1280	Sept. 2004	Ningera, SP	-2.73997	141.4407	*Citrus* x *aurantiifolia*
MPW1279	Sept. 2004	Ningera, SP	-2.73997	141.4407	*Citrus japonica*
MPW1285	Sept. 2004	Blackwater, SP	-2.77392	141.3893	*Citrus* sp.
MPW1291	Sept. 2004	Dapu, SP	-2.70808	141.3271	*Citrus* sp.
MPW1291	Sept. 2004	Dapu, SP	-2.70808	141.3271	*Citrus* sp.
MPW1301	Sept. 2004	Vanimo, SP	-2.7049	141.3037	*Citrus* x *aurantium*
MPW1301	Sept. 2004	Vanimo, SP	-2.7049	141.3037	*Citrus* x *aurantium*
MPW1298	Sept. 2004	Vanimo, SP	-2.70327	141.3038	*Citrus* x *limon*
MPW1288	Sept. 2004	Wusipi, SP	-2.72477	141.3406	*Citrus* x *aurantium*
MPW1300	Sept. 2004	Vanimo, SP	-2.70378	141.3039	*Citrus* x *aurantiifolia*
MPW1289	Sept. 2004	Wusipi, SP	-2.72515	141.3407	*Citrus* x *limon*
MPW1289	Sept. 2004	Wusipi, SP	-2.72515	141.3407	*Citrus* x *limon*
MPW1304	Sept. 2004	Dapu, SP	-2.82507	141.3282	*Citrus* x *limon*
MPW1311	Sept. 2004	Vanimo, SP	-2.67782	141.3083	*Citrus japonica*
MPW1325	Sept. 2004	Vanimo, SP	-2.68443	141.305	*Citrus* sp.
MPW1313	Sept. 2004	Vanimo, SP	-2.67738	141.3045	*Citrus* sp.
MPW1275	Sept. 2004	Ningera, SP	-2.72715	141.3856	*Citrus japonica*
MPW1294	Sept. 2004	Dapu, SP	-2.70823	141.3281	*Citrus* sp.
MPW1331	Sept. 2004	Aitape, SP	-3.15133	142.3658	*Citrus reticulata*
MPW1327	Sept. 2004	Aitape, SP	-3.17647	142.3989	*Citrus reticulata*
MPW1328	Sept. 2004	Aitape, SP	-3.12507	142.3087	*Citrus reticulata*
MPW1329	Sept. 2004	Aitape, SP	-3.12507	142.3087	*Citrus* x *aurantium*
MPW1335	Sept. 2004	Ituli, SP	-3.02465	141.1379	*Citrus reticulata*
MPW1344	Sept. 2004	Yako, SP	-2.61893	141.0909	*Citrus* sp.
MPW1343	Sept. 2004	Mushu, SP	-2.62765	141.1018	*Citrus* sp.
MPW1338	Sept. 2004	Wutung, SP	-2.60645	141.0055	*Citrus* x *aurantiifolia*
MPW1340	Sept. 2004	Wutung, SP	-2.60645	141.0055	*Citrus* x *aurantiifolia*
JR174	Nov. 2006	Lowan ESP	-3.38528	143.1781	*Citrus* sp.
JR183	Nov. 2006	Aitape, SP	-3.13194	142.3356	*Citrus* sp.
JR182	Nov. 2006	Wewak, ESP	-3.54826	143.627	*Citrus* sp.
JR180	Nov. 2006	Moem, ESP	-3.56612	143.6974	*Citrus* sp.
JR206	Nov. 2006	Vanimo, SP	-2.67861	141.3072	*Citrus* sp.
JR207	Nov. 2006	Vanimo, SP	-2.67861	141.3072	*Citrus* sp.
JR205	Nov. 2006	Vanimo, SP	-2.66889	141.2561	*Citrus* sp.
JR199	Nov. 2006	Wutung, SP	-2.6692	141.3081	*Citrus* sp.

JR190	Nov. 2006	Yako, SP	-2.63361	141.185	*Citrus* sp.
JR191	Nov. 2006	Yako, SP	-2.63361	141.185	*Citrus* sp.
JR194	Nov. 2006	Wutung, SP	-2.61361	141.0075	*Citrus* sp.
JR193	Nov. 2006	Wutung, SP	-2.61361	141.0075	*Citrus reticulata*
JR198	Nov. 2006	Wutung, SP	-2.60509	141.0017	*Citrus* sp.
JR213	Nov. 2006	Wusipi, SP	-2.73	141.345	*Citrus* sp.
JR211	Nov. 2006	Samboi, SP	-3.05583	141.1683	*Citrus* sp.
JR217	Nov. 2006	Amanab, SP	-3.59083	141.202	*Citrus* x *aurantiifolia*
JR226	Nov. 2006	Telefomin, SP	-5.15972	141.6183	*Citrus* sp.
RID4795	Oct. 2007	Moem, ESP	-3.5663	143.6973	*Citrus* sp.
RID4792	Oct. 2007	Moem, ESP	-3.56652	143.6973	*Citrus* sp.
RID4818	Oct. 2007	Haniak, ESP	-3.74428	143.5013	*Citrus reticulata*
RID4837	Oct. 2007	Aitape, SP	-3.57365	142.6172	*Citrus japonica*
RID4838	Oct. 2007	Aitape, SP	-3.57365	142.6172	*Citrus maxima*
RID4845	Oct. 2007	Ulau, SP	-3.30952	142.7923	*Citrus japonica*
RID4852	Oct. 2007	Aitape, SP	-3.1679	142.3595	*Citrus* x *aurantium*
RID4862	Oct. 2007	Wauningi, SP	-3.20363	142.1957	*Citrus x limon*
RID4873	Oct. 2007	Aitape, SP	-3.12562	142.3468	*Citrus* x *aurantium*
RID5021	June 2008	Daru, WP	-9.0737	143.212	*Citrus reticulata*
RID5151	June 2008	Tabubil, WP	-5.29	141.2333	*Citrus reticulata*
RID5381	June 2009	Vanimo, SP	-2.68583	141.3039	*Citrus japonica*
RID5383	June 2009	Vanimo, SP	-2.69023	141.303	*Citrus japonica*
RID5408	June 2009	Vanimo, SP	-2.68471	141.3062	*Citrus japonica*
RID5406	June 2009	Vanimo, SP	-2.68471	141.3062	*Citrus* x *limon*
RID5400	June 2009	Lido SP	-2.6626	141.277	*Citrus japonica*
RID5397	June 2009	Lido SP	-2.6633	141.2767	*Citrus* sp.
RID5405	June 2009	Lido SP	-2.66554	141.2682	*Citrus* sp.
RID5402	June 2009	Lido SP	-2.66171	141.2769	*Citrus* x *aurantiifolia*
RID5393	June 2009	Lido SP	-2.664	141.2262	*Citrus* x *aurantiifolia*
RID5396	June 2009	Lido SP	-2.664	141.2262	*Citrus japonica*
RID5429	June 2009	Yako, SP	-2.63371	141.194	*Citrus* x *limon*
RID5466	June 2009	Telefomin, SP	-5.13764	141.6251	*Citrus japonica*
RID5457	June 2009	Vanimo, SP	-2.69126	141.2981	*Citrus japonica*
RID5460	June 2009	Vanimo, SP	-2.69126	141.2981	*Citrus* x *limon*
RID5458	June 2009	Vanimo, SP	-2.69159	141.2995	*Citrus maxima*
RID5478	June 2009	Pasi, SP	-2.73659	141.342	*Citrus* x *limon*
RID5491	June 2009	Dapu, SP	-2.70812	141.3268	*Citrus japonica*
RID5469	June 2009	Blackwater, SP	-2.77386	141.3907	*Citrus* x *limon*
RID5489	June 2009	Warastone, SP	-2.71382	141.3389	*Citrus japonica*
RID5486	June 2009	Warastone, SP	-2.71361	141.3386	*Citrus* x *limon*
RID5483	June 2009	Wusipi, SP	-2.72515	141.3407	*Citrus* x *limon*
RID5493	June 2009	Vanimo, SP	-2.68112	141.3067	*Citrus* x *limon*

RID5505	June 2009	Hogi, ESP	-3.4133	143.3866	*Citrus japonica*
RID5514	June 2009	Yangorum, ESP	-3.65638	143.2967	*Citrus x limon*
RID5525	June 2009	Wewak, ESP	-3.5748	143.6169	*Citrus maxima*
RID5522	June 2009	Kongen, ESP	-3.61024	143.7163	*Citrus japonica*
RID5629	June 2009	Alotau, MBP	-10.3082	150.4484	*Citrus* sp.
RID6102	June 2009	Nadzab, MoP	-6.6833	147.1	*Citrus* sp.
RID6168	June 2009	Dar MS, SHP	-6.24529	143.552	*Citrus reticulata*
RID6252	June 2009	Samagos, WP	-6.09361	141.3072	*Citrus reticulata*
RID6292	June 2009	Wando, WP	-8.88907	141.2593	*Citrus maxima*
RID6296	June 2009	Kamusi, WP	-7.42442	143.1228	*Citrus* x *limon*
RID6453	Oct. 2012	Wewak, ESP	-3.7196	143.5963	*Citrus reticulata*
RID6465	Oct. 2012	Wogan, SP	-3.12503	142.1309	*Citrus reticulata*
RID6462	Oct. 2012	Poro, SP	-3.19967	142.1889	*Citrus reticulata*
RID6473	Nov. 2012	Wutung, SP	-2.62984	141.1354	*Citrus maxima*
RID6472	Nov. 2012	Wutung, SP	-2.60643	141.0055	*Citrus reticulata*
RID6480	Nov. 2012	Passi, SP	-2.7356	141.3414	*Citrus maxima*
RID6495	Nov. 2012	Amanab, SP	-3.58576	141.216	*Citrus x limon*
RID6500	Nov. 2012	Lido, SP	-2.66436	141.2774	*Citrus* sp.
RID6501	Nov. 2012	Lido, SP	-2.66436	141.2774	*Citrus* x *limon*
RID6502	Nov. 2012	Lido, SP	-2.66436	141.2774	*Citrus* x *limon*
RID6537	May 2013	Daru, WP	-9.0737	143.212	*Citrus maxima*
RID6652	May 2014	Kabiufa, EHP	-5.99062	145.3783	*Citrus reticulata*
RID6639	May 2014	Osaro, EHP	-5.97596	145.2777	*Citrus reticulata*
RID6656	May 2014	Ku, CP	-6.04516	145.018	*Citrus* sp.
RID6675	June 2014	Minj, JP	-5.88307	144.6829	*Citrus reticulata*
RID7025	May 2015	Ningerum, WP	-5.72144	141.105	*Citrus reticulata*
RID7041	May 2015	Aiambak, WP	-7.34794	141.2674	*Citrus* sp.
RID7067	May 2015	Daru, WP	-9.06938	143.2132	*Citrus* x *aurantium*
MA05	March 2017	Buji, WP	-9.15089	142.2355	*Citrus* sp.
MA13	March 2017	Bula, WP	-9.12803	141.3415	*Citrus* sp.
MA09	March 2017	Jarai, WP	-9.19783	141.5841	*Citrus* sp.
LMJ1220	Sept. 2018	Tinputz, AROB	-5.5552	155.0079	*Citrus* sp.

[A]AROB: Autonomous Region of Bougainville, CP: Chimbu Province, EHP: Eastern Highlands Province, ESP: East Sepik Province, JP: Jiwaka Province, MaP: Madang Province, MoP: Morobe Province, MBP: Milne Bay Province, SP: Sandaun Province, SHP: Southern Highlands Province, WP: Western Province

Each sample consisted of desiccated petioles and midribs and DNA was extracted, using either Qiagen DNeasy plant or Bioline Isolate II Plant DNA kits. They were tested in a multiplex PCR reaction using the primers A2/J5 [6] to detect '*Ca*. Liberibacter' and primers rP1/fD1 [7] which amplifying 16SrDNA of other bacteria present in DNA preparations to

verify the PCR-competency of each individual reaction mixture. GPS coordinates are provided to indicate breadth of coverage across locations intensively surveyed.

Table S6. Palm samples collected from outside known BCS affected areas that indexed negative for phytoplasma in PCR testing

Collection No.	Approx. date	Location[A]	Latitude	Longitude	Palm
RID4831	Oct. 2007	Aitape, SP	-3.57365	142.6172	*Cocos nucifera*
RID4834	Oct. 2007	Aitape, SP	-3.57365	142.6172	*Cocos nucifera*
RID5114	June 2008	Tarakbits, WP	-5.60925	141.0439	*Areca catechu*
RID5581	Oct. 2009	Gusap, MoP	-5.95663	145.8808	*Cocos nucifera*
RID5575	Oct. 2009	Muzing, MoP	-6.36744	146.3154	*Cocos nucifera*
RID5885	Nov. 2010	Buka Is. AROB	-5.4181	154.6259	*Cocos nucifera*
RID5988	Nov. 2010	Sanakoba, Bougainville Is., AROB	-5.45417	154.7607	*Areca catechu*
RID6254	Oct. 2011	Manoia, WP	-5.95306	141.3592	*Metroxylon sagu*
RID6257	Oct. 2011	Manoia, WP	-5.95306	141.3592	*Metroxylon sagu*
RID6494	Nov. 2012	Yapsi, SP	-4.62881	141.0957	*Cocos nucifera*
RID6459	Nov. 2012	Poro, SP	-3.19889	142.1875	*Cocos nucifera*
RID6712	May 2014	Six mile, Port Moresby, NCD	-5.91656	143.9226	*Elaeis guineensis*
RID6713	May 2014	Six mile, Port Moresby, NCD	-5.91656	143.9226	*Elaeis guineensis*
RID7032	May 2015	Ningerum, WP	-5.71871	141.1093	*Hydriastele costata*
RID7050	May 2015	Debepari, WP	-6.30835	141.9056	*Hydriastele costata*
RID7054	May 2015	Morehead, WP	-8.71271	141.6422	*Cocos nucifera*
RID7048	May 2015	Debepari, WP	-6.30835	141.9056	*Cocos nucifera*
RID7049	May 2015	Debepari, WP	-6.30835	141.9056	*Cocos nucifera*
GUN-WG S1D1	Sept. 2016	Gunjangi, ESP			*Cocos nucifera*
GUN-WG S1D2	Sept. 2016	Gunjangi, ESP			*Cocos nucifera*
NAN-AD S3D1	Sept. 2016	Nanaha, ESP			*Cocos nucifera*
NAN-AD S3D1	Sept. 2016	Nanaha, ESP			*Cocos nucifera*
MA07	March 2017	Bula, WP	-9.12802	141.3413	*Cocos nucifera*
MA45	March 2017	Mabaduan, WP	-9.27773	142.7341	*Cocos nucifera*
MA38	March 2017	Mari, WP	-9.19492	141.7041	*Cocos nucifera*
RID7702	Nov. 2017	Brahman, MaP	-5.75613	145.3625	*Cocos nucifera*
RID7703	Nov. 2017	Brahman, MaP	-5.75608	145.3626	*Cocos nucifera*
RID7704	Nov. 2017	Brahman, MaP	-5.75621	145.3633	*Cocos nucifera*
RID7705	Nov. 2017	Brahman, MaP	-5.75662	145.3633	*Cocos nucifera*
RID7706	Nov. 2017	Brahman, MaP	-5.75643	145.3634	*Cocos nucifera*
RID7707	Nov. 2017	Brahman, MaP	-5.75651	145.3633	*Cocos nucifera*
RID7732	Nov. 2017	Maprik, ESP	-3.67706	143.8291	*Cocos nucifera*

RID7733	Nov. 2017	Maprik, ESP	-3.67706	143.8291	*Cocos nucifera*
RID7749	Nov. 2017	Angoram, ESP	-4.05784	144.0704	*Cocos nucifera*
RID7749	Nov. 2017	Angoram, ESP	-4.05784	144.0704	*Hydriastele costata*
LMJ1216	Sept. 2018	Teperoi, Bougainville Is. AROB	-5.90623	155.2787	*Cocos nucifera*
LMJ1253	Sept. 2018	Kiriwa, Bougainville Is., AROB	-6.72416	155.4793	*Cocos nucifera*
LMJ1230	Sept. 2018	Toiomanapu, Bougainville Is., AROB	-6.45874	155.855	*Cocos nucifera*
LMJ1227	Sept. 2018	Toiomanapu, Bougainville Is., AROB	-6.45849	155.8554	*Cocos nucifera*
WNB1*	April 2018	Makuar, WNBP			*Cocos nucifera*
WNB2*	April 2018	Makuar, WNBP			*Cocos nucifera*
WNB3A*	April 2018	Airagilpua, WNBP			*Cocos nucifera*
WNB3B*	April 2018	Airagilpua, WNBP			*Cocos nucifera*
WNB4*	April, 2018	Almango, WNBP			*Cocos nucifera*
WNB5*	April 2018	Ongaea, WNBP			*Areca catechu*
WNB6*	April 2018	Ongaea, WNBP			*Areca catechu*
WNB7*	April 2018	Ongaea, WNBP			*Cocos nucifera*
WNB8*	April 2018	Gloucester, WNBP			*Cocos nucifera*
WNB9*	April 2018	Walai, WNBP			*Cocos nucifera*
WNB10*	April 2018	Dagi, WNBP			*Cocos nucifera*
WNB11*	April 2018	Dagi, WNBP			*Areca catechu*
WNB13*	April 2018	Sarakolok, WNBP			*Cocos nucifera*
WNB14*	April 2018	Sarakolok, WNBP			*Areca catechu*
MA289*	Nov. 2018	Gidobada, CP			*Cocos nucifera*
MA287*	Nov. 2018	Loga Makana, CP			*Cocos nucifera*
MA284*	Nov. 2018	Saroa Keina, CP			*Cocos nucifera*
MA286*	Dec. 2018	Ganai, CP			*Hydriastele costata*
MA285*	Dec. 2018	N/A, CP			*Areca catechu*
MA288*	Dec. 2018	N/A, CP			*Areca catechu*
MA290*	Dec. 2018	N/A, CP			*Areca catechu*
MA388*	Nov. 2019	Tugiyag, Rai Coast, MaP			*Cocos nucifera*
MA342*	Nov. 2019	Dumbal, Rai Coast,MaP			*Cocos nucifera*
MA341*	Nov. 2019	Ganglau, Rai Coast, MaP			*Areca catechu*
MA347*	Nov. 2019	Ganglau, Rai Coast, MaP			*Areca catechu*
LS33*	Nov. 2019	Namatanai, NIP			*Cocos nucifera*

[A]AROB: Autonomous Region of Bougainville, CP: Central Province, ESP: East Sepik Province, MaP: Madang Province, MoP: Morobe Province, NCD: National Capital District, NIP: New Ireland Province, SP: Sandaun Province, WNBP: West New Britain Province, WP: Western Province

Each sample consisted of internal lower trunk or meristematic tissues collected and indexed by nested PCR in Australia as described in [79]. Samples labelled '*' were indexed in New Zealand using generic phytoplasma primers by the New Zealand Ministry for Primary Industries Plant Health and Environment Laboratory. GPS coordinates are provided to indicate breadth of coverage across locations intensively surveyed. GPS coordinates are provided, where available, to indicate breadth of coverage across locations intensively surveyed.

References

1. Mowat, W.P.; Dawson, S. Detection and identification of plant viruses by ELISA using crude sap extracts and unfractionated antisera. *J. Virol. Methods* **1987**, *15*, 233–247.
2. Marie–Jeanne, V.; Ioose, R.; Peyre, J.; Alliot, B.; Signoret, P. Differentiation of Poaceae potyviruses by reverse transcription-polymerase chain reaction and restriction analysis. *J. Phytopathol.* **2000**, *148*, 141–151.
3. Sharman, M.; Thomas, J.E.; Dietzgen, R.G. Development of a multiplex immunocapture PCR with colourimetric detection for viruses of banana. *J. Virol. Methods* **2000**, *89*, 75–88.
4. Clark, M.F.; Adams, A.N. Characteristics of the microplate method of enzyme-linked immunosorbent assay for the detection of plant viruses. *J. Gen. Virol.* **1977**, *34*, 475–483.
5. Smith, G.R.; Van de Velde, R. Detection of sugarcane mosaic virus and Fiji disease virus using the polymerase chain reaction. *Plant Dis.* **1994**, *78*, 557–561.
6. Hocquellet, A.; Toorawa, P.; Bové, J.-M.; Garnier, M. Detection and identification of the two Candidatus Liberobacter species associated with citrus huanglongbing by PCR amplification of ribosomal protein genes of the i operon. *Mol. Cell. Probes* **1999**, *13*, 373–379.
7. Weisburg, W.G.; Barns, S.M.; Pelletier, D.A.; Lane, D.J. 16S ribosomal DNA amplification for phylogenetic study. *J. Bacteriol.* **1991**, *173*, 697–703.
